# Laparoscopic *versus* open liver resection in patients aged at least 80 years: retrospective propensity score-matched cohort study

**DOI:** 10.1093/bjsopen/zraf102

**Published:** 2025-11-28

**Authors:** Concepción Gómez-Gavara, Zenichi Morise, Victor López-López, Christoph Kuemmerli, Daniel Esono, Kazuharu Igarashi, Kohei Mishima, Akishige Kanazawa, Shogo Tanaka, Shoji Kubo, Satoshi Nemoto, Goro Honda, Kazuteru Monden, Masaki Ueno, Yasuhito Iwao, Naoto Gotohda, Masashi Kudo, Hiroyuki Nitta, Satoshi Amano, Rafael Díaz-Nieto, Alex Gordon-Weeks, Serena Langella, Alessandro Ferrero, Yuichiro Otsuka, Hironori Kaneko, Riccardo Boetto, Umberto Cillo, Daniel D’Souza, Pablo E Serrano, Giammauro Berardi, Marco Angrisani, Giuseppe Maria Ettorre, Parissa Tabrizian, Allen Yu, Brian K P Goh, Takuya Minagawa, Osamu Itano, Daisuke Asano, Minoru Tanabe, Marcello Di Martino, Elena Martín-Pérez, Simone Famularo, Elisa Paoluzzi Tomada, Guido Torzilli, Jaime Arthur Pirola Krüger, Paulo Herman, Mario Giuffrida, Ramon Charco, Mikel Gastaca, Waclaw Holowko, Stephanie Truant, Kit-Man Ho, Kai-Chi Cheng, Rafael José Maurette, Laura-Ann Blatt, Tatiana Belda, Yuta Abe, Shuichiro Uemura, Go Wakabayashi

**Affiliations:** Hepatopancreatobiliary and Transplantation Surgery Service, Vall d’Hebron University Hospital, Barcelona, Spain; Department of Surgery, Fujita Health University School of Medicine Okazaki Medical Center, Okazaki, Aichi, Japan; Department of General, Visceral and Transplantation Surgery, Clinic and University Hospital Virgen de La Arrixaca, IMIB-ARRIXACA, El Palmar, Spain; Department of Surgery, Clarunis University Center for Gastrointestinal and Liver Disease, St Clara Hospital and University Hospital, Basel, Switzerland; Department of Information and Communications Technologies, Universitat Pompeu Fabra, Barcelona, Spain; Department of Surgery, Ageo Central General Hospital, Ageo, Japan; Department of Surgery, Ageo Central General Hospital, Ageo, Japan; Department of Hepato-Biliary-Pancreatic Surgery, Osaka City General Hospital, Osaka, Japan; Department of Hepato-Biliary-Pancreatic Surgery, Osaka City University Graduate School of Medicine, Osaka, Japan; Department of Hepato-Biliary-Pancreatic Surgery, Osaka City University Graduate School of Medicine, Osaka, Japan; Department of Surgery, Institute of Gastroenterology, Tokyo Women’s Medical University, Tokyo, Japan; Department of Surgery, Institute of Gastroenterology, Tokyo Women’s Medical University, Tokyo, Japan; Department of Surgery, Fukuyama City Hospital, Fukuyama, Japan; Department of Surgery, Wakayama Medical University, Wakayama, Japan; Department of Surgery, Ohta-Nishinouchi Hospital, Kōriyama, Japan; Department of Surgery, National Cancer Center Hospital East, Chiba, Japan; Department of Surgery, National Cancer Center Hospital East, Chiba, Japan; Department of Surgery, Iwate Medical University, Iwate, Japan; Department of Surgery, Iwate Medical University, Iwate, Japan; Department of Surgery, Liverpool University Hospital, Liverpool, UK; Department of Surgery, Liverpool University Hospital, Liverpool, UK; S Chirurgia Generale e Oncologica, Ospedale Mauriziano, Turín, Italy; S Chirurgia Generale e Oncologica, Ospedale Mauriziano, Turín, Italy; Department of Surgery, Toho University Faculty of Medicine, Tokyo, Japan; Department of Surgery, Toho University Faculty of Medicine, Tokyo, Japan; Department of Surgery, Oncology and Gastroenterology, Hepatobiliary Surgery and Liver Transplantation Unit, Padova University Hospital, Padova, Italy; Department of Surgery, Oncology and Gastroenterology, Hepatobiliary Surgery and Liver Transplantation Unit, Padova University Hospital, Padova, Italy; Department of Surgery McMaster University, Hamilton, Ontario, Canada; Department of Surgery McMaster University, Hamilton, Ontario, Canada; Azienda Ospedaliera San Camillo Forlanini, General Surgery and Transplant Unit, Rome, Italy; Azienda Ospedaliera San Camillo Forlanini, General Surgery and Transplant Unit, Rome, Italy; Azienda Ospedaliera San Camillo Forlanini, General Surgery and Transplant Unit, Rome, Italy; Mount Sinai Liver Cancer Program, Tisch Cancer Institute, Icahn School of Medicine at Mount Sinai, New York, New York, USA; Mount Sinai Liver Cancer Program, Tisch Cancer Institute, Icahn School of Medicine at Mount Sinai, New York, New York, USA; Department of Hepatopancreatobiliary and Transplant Surgery, Singapore General Hospital and National Cancer Centre, Singapore, Singapore; Department of Hepato-Biliary-Pancreatic and Gastrointestinal Surgery, School of Medicine, International University of Health and Welfare, Chiba, Japan; Department of Hepato-Biliary-Pancreatic and Gastrointestinal Surgery, School of Medicine, International University of Health and Welfare, Chiba, Japan; Department of Hepatobiliary and Pancreatic Surgery, Graduate School of Medicine, Tokyo Medical and Dental University, Tokyo, Japan; Department of Hepatobiliary and Pancreatic Surgery, Graduate School of Medicine, Tokyo Medical and Dental University, Tokyo, Japan; Hepatopancreatobiliary Unit, Department of General and Digestive Surgery, Hospital Universitario La Princesa, Instituto deInvestigación Sanitaria Princesa (IIS-IP), Universidad Autónoma de Madrid (UAM), Madrid, Spain; Hepatopancreatobiliary Unit, Department of General and Digestive Surgery, Hospital Universitario La Princesa, Instituto deInvestigación Sanitaria Princesa (IIS-IP), Universidad Autónoma de Madrid (UAM), Madrid, Spain; Department of Biomedical Sciences, Humanitas University, Pieve Emanuele, Milan, Italy; Department of Biomedical Sciences, Humanitas University, Pieve Emanuele, Milan, Italy; Department of Biomedical Sciences, Humanitas University, Pieve Emanuele, Milan, Italy; Department of Gastroenterology, Hospital das Clinicas, University of São Paulo Medical School, São Paulo, Brazil; Department of Gastroenterology, Hospital das Clinicas, University of São Paulo Medical School, São Paulo, Brazil; General Surgery Unit, Parma University Hospital, Parma, Italy; Hepatopancreatobiliary and Transplantation Surgery Service, Vall d’Hebron University Hospital, Barcelona, Spain; Hepatobiliary Surgery and Liver Transplant Unit, Cruces University Hospital BioCruces Health Research Institute, Vizcaya, Spain; Department of General, Transplant and Liver Surgery, Medical University of Warsaw, Warsaw, Poland; Department of Digestive Surgery and Liver Transplantation, University Hospital C. Huriez, Lille, France; Department of Surgery, Kwong Wah Hospital, Hong Kong, China; Department of Surgery, Kwong Wah Hospital, Hong Kong, China; General Surgery Department, British Hospital of Buenos Aires, Buenos Aires, Argentina; Department of General, Visceral and Thoracic Surgery, Surgical Oncology, Klinikum Saarbruecken, Saarbruecken, Germany; Department of Surgery, Verge dels Lliris Hospital, Alcoy, Spain; Department of Surgery, Keio University School of Medicine, Keio University Hospital, Tokyo, Japan; Department of Surgery, Keio University School of Medicine, Keio University Hospital, Tokyo, Japan; Department of Surgery, Ageo Central General Hospital, Ageo, Japan

**Keywords:** geriatric population, older adults, minimally invasive surgery, complications, matching score, Iwate score

## Abstract

**Background:**

Laparoscopic liver resection has been associated with less morbidity than, and similar global outcomes to, open liver resection. There is no robust evidence that these outcomes lead to similar clinical outcomes in patients aged over 80 years. The aim of this study was to analyse the short-term outcomes between open and laparoscopic liver resection in patients over 80 years old.

**Methods:**

A retrospective analysis was undertaken. The study population comprised patients aged ≥ 80 years who underwent laparoscopic or open liver resection between January 2014 and December 2019, and who presented with resectable malignant tumours. The primary outcome was postoperative morbidity, according to Dindo-Clavien grading. Cox regression models were used to compute hazard ratios and 95% confidence intervals. Propensity score matching (1 : 1) was performed to balance the two groups according to independent prognostic factors for morbidity.

**Results:**

A total of 988 patients were analysed from 34 centres (16 from Asia, 14 from Europe and 4 from America): 487 in the open group and 501 in the laparoscopic group. Independent risk factors associated with severe morbidity were the open approach (hazard ratio 1.59, 95% confidence interval 1.19 to 2.11; *P <* 0.001), Charlson Co-morbidity Index score > 7 (HR 1.69, 1.26 to 2.27; *P <* 0.001), more than one resected tumour (hazard ratio 1.55, 1.13 to 2.11; *P =* 0.006), major hepatectomy (hazard ratio 1.86, 1.22 to 2.83; *P =* 0.003), and Iwate score ≥ 7 (hazard ratio 1.43, 1.02 to 2.01; *P =* 0.03). Before propensity score matching, severe morbidity, length of intensive care unit stay, 90-day mortality, length of hospital stay, and readmission were better in the laparoscopic group (*P* < 0.050). These observations were confirmed after propensity score matching.

**Conclusion:**

The laparoscopic approach is a safe procedure for elderly patients, with better morbidity and mortality outcomes than the open approach, and should be considered as a default option.

## Teaser

This is the largest study to compare laparoscopic *versus* open hepatectomy in patients aged over 80 years. It is an international multicentre study including 988 patients from 34 centres, 487 (49.3%) in the open group and 501 (50.7%) in the laparoscopic group. It has shown that open hepatectomy in patients over 80 years old is associated with an increased risk of morbidity and 90-day mortality.

## Introduction

Well known benefits of laparoscopy include decreased blood loss, postoperative pain, and length hospital of stay; improved mobilization and quicker return to normal activity; and fewer pulmonary, thrombotic, and abdominal wall complications^1,2^. It has been questioned whether elderly patients can reap the same benefits from laparoscopic surgery in the liver surgery setting^3^. Whether it is better to have a short open operation or a longer laparoscopic procedure is under debate as it depends on the impact of the physiological requirements of the pneumoperitoneum^4^. Thanks to recent advancements in intraoperative anaesthetic care, there is no evidence that longer operating time and pneumoperitoneum can lead to worse clinical outcomes in elderly patients^5^. For patients aged between 70 and 79 years, laparoscopic liver resection is still used, whereas the open approach is most commonly preferred for patients aged > 80 years^6–8^. Several publications and meta-analyses^9–15^ have reported contrasting results about the benefits of minimally invasive surgery in elderly patients aged > 80 years, and in most studies octogenarians were included only in the open group. Although there is still a tendency to perform open surgery for elderly patients in Europe, the laparoscopic approach is preferred in Asia, even among octogenarians.

The primary aim of this study was to establish whether the laparoscopic or open approach is more appropriate for elderly patients regarding morbidity and short-term outcomes.

## Methods

### Study population

Data from patients aged ≥ 80 years undergoing laparoscopic or open liver resection for malignant liver tumours between January 2014 and December 2019 were collected from a dedicated multi-institutional database (34 centres) after institutional approval at each centre. Inclusion criteria were: patients aged > 80 years with a resectable malignant liver tumour (colorectal liver metastases, hepatocellular carcinoma, cholangiocarcinoma or any other type). Exclusion criteria were: patients who had emergency surgery, simultaneous resection of other organs, distant metastases on abdominal or thoracic computed tomography, previous surgical or local ablative tumour treatment, or combined vascular, biliary, or lymphadenectomy procedures whenever performed. All participating centres had experience of > 100 hepatobiliary resections during the study period. Patients whose procedure was converted from a laparoscopic to an open approach were considered in the laparoscopic group.

### Variables

Clinical preoperative variables included demographics, American Society of Anesthesiologists grade^16^, co-morbidities (Charlson Co-morbidity Index^17^), performance status^18^, underlying liver disease, and preoperative tumour features. Preoperative serum values assessed were albumin level, total bilirubin level, prothrombin time, and platelet count. Liver disease was defined as severe, if cirrhosis and portal hypertension were present with a history of variceal bleeding; moderate, if cirrhosis and portal hypertension were present without a history of variceal bleeding; and mild, if chronic hepatitis or cirrhosis was present without portal hypertension. Portal hypertension was defined by a hepatic venous pressure gradient before surgery of > 10 mmHg or a platelet count of ≤ 100 000/ml^19^.

Preoperative difficulty was evaluated according to the Iwate scoring system^1^.

Each specimen was subjected to pathological assessment, with recording of the type, size, and number of tumours. Operative variables included the type of hepatectomy, and the need for vascular clamping, intraoperative blood transfusion, hand assistance, and/or conversion to an open approach. Major and extended major resections were defined as resection of at least three or at least five Couinaud segments, respectively^20^. Intraoperative blood transfusion was defined as the infusion of packed red blood cells in the operating room. Other blood products including fresh frozen plasma, cryoprecipitate, and platelets were not included in this definition. Liver failure was defined according to 50–50 criteria^21^.

Mortality and morbidity were measured within 90 days of surgery. Morbidity was graded according to the Comprehensive Complication Index (CCI)^22^. Clavien–Dindo grading^22^ was also evaluated. Severe morbidity was defined as any complication with a Clavien–Dindo grade > III. A CCI ≥26.2, as described by Yamashita *et al.*^23^, was also used as reference standard to describe relevant postoperative complications. Follow-up and long-term clinical monitoring were carried out after surgery according to the custom of each centre.

### Reporting data

Patient data were recorded by the local principal investigator. The study was registered in Research Registry (10218). Patients were coded anonymously by a numeric code, and a subject identification code list was made and stored separately by the local investigator, who safeguarded the code.

### Ethical considerations

The study was conducted in accordance with the principles of the Declaration of Helsinki, and the Medical Research Involving Human Subjects Act of the World Medical Association^24^. Regulation 2016/679 of the European Parliament and of the council on the protection of individuals regarding the processing of personal data and on the free movement of such data, and the Organic Law 3/2018 of 5 December on the Protection of Personal Data and guarantee of digital rights.

The study protocol and data protection officer were approved by the local ethics committee of each hospital and by the coordinator University Hospital (PR(AG)188/2021).

### Statistical analysis

Qualitative variables are expressed as frequencies and percentages, and were analysed by χ^2^ or Fisher’s exact test when the frequency was low, adding a study of standardized residuals to see the directionality of the associations. Quantitative variables are presented as mean(standard deviation, s.d.) if they followed a normal distribution, and as median (interquartile range, i.q.r.) otherwise, with analysis using Student’s *t* test (grouped *t* test) or the non-parametric Mann–Whitney *U* test, respectively. Two-sided *P* < 0.050 was considered to indicate statistical significance.

Patients from the open and laparoscopic groups were matched using propensity score matching (PSM) as described by Rubin, Thomas, and Rosenbaum^25,26^. To balance the two populations, PSM 1 : 1 nearest-neighbour matching was used. PSM was done with a multivariable logistic regression model using confounders related to both the exposure and the outcome selected *a priori* based on background knowledge as the independent variable. Baseline variables with prognostic relevance as determined in a multivariable logistic model with *P* ≤ 0.050 (Charlson Co-morbidity Index, number of resected tumours, major hepatectomy, and Iwate score) were included. Exact matching was forced for the independent risk factors. The caliper width was set to 0.1. After matching, all standardized mean differences for the co-variates were below 0.2, indicating good balance.

Statistical analysis was performed with R version 4.0.4 (R Foundation for Statistical Computing, Vienna, Austria).

## Results

After excluding 60 patients because they had at least 1 missing value among the variables used for PSM, a total of 988 patients were analysed from 34 centres (16 from Asia, 14 from Europe and 4 from the Americas). There were 487 (49.3%) in the open group and 501 (50.7%) in the laparoscopic group. *Table 1* summarizes the main characteristics of the study population. *Figure S1* is a histogram of the patients’ distribution per centre.

**Table 1:** Patient characteristics in open and laparoscopic groups before propensity score matching

	Open group (*n* = 487)	Laparoscopic group (*n* = 501)	*P*§
**Patient characteristics**			
Age (years), median (i.q.r.)	82 (81–83)	82 (81–84)	0.87¶
Sex			0.33
Male	315 (64.6%)	328 (65.4%)	
Female	172 (35.4%)	173 (34.6%)	
ASA fitness grade, median (i.q.r.)	III (II–III)	II (II–III)	0.04¶
BMI ≥ 30 kg/m^2^	62 (12.7%)	46 (9.1%)	0.05
Charlson Co-morbidity Index score, median (i.q.r.)^17^	7 (5–7)	7 (5–9)	0.73¶
Performance status > 1^18^	109 (22.3%)	109 (21.7%)	0.81
**Tumour characteristics**			
Tumour location (liver segment)			0.01
I	11 (2.2%)	5 (0.9%)	
II–IV	174 (35.7%)	195 (38.9%)	
V–VI	118 (24.1%)	153 (30.5%)	
VII–VIII	184 (38%)	148 (29.7%)	
No. of tumours, median (i.q.r.)	1 (1–2)	1 (1–1)	< 0.001¶
Type of primary tumour			< 0.001
Hepatocellular carcinoma	190 (39.1%)	260 (51.8%)	
Colorectal liver metastases	179 (36.7%)	167 (33.3%)	
Cholangiocarcinoma	76 (15.6%)	31 (6.2%)	
Others	42 (8.6%)	43 (8.6%)	
**Surgical characteristics**			
Type of resection			<0.001
Partial	156 (32%)	251 (50%)	< 0.001
Left lateral sectionectomy	22 (4.5%)	41 (8.2%)	0.01
Segmentectomy	113 (23.2%)	125 (25%)	0.52
Sectionectomy and more	196 (40.3%)	84 (16.8%)	< 0.001
Wedge resection	191 (39.2%)	236 (47.1%)	0.01
Anatomical resection	319 (65.5%)	239 (47.7%)	< 0.001
Major hepatectomy	187 (38.4%)	76 (15.1%)	< 0.001
Iwate score ≥ 7	245 (50%)	164 (32.7%)	< 0.001
**Outcomes**			
Total Pringle time (min), mean(s.d.)	24.4(39.2)	39.8(53.6)	< 0.001#
Intraoperative blood loss (ml), mean(s.d.)	517(552.3)	251(422)	< 0.001#
Intraoperative blood transfusion	93 (19%)	43 (8.5%)	< 0.001
Conversion to open surgery	–	32 (6.3%)	
Duration of operation (min), mean(s.d.)	257.9(125.7)	265.2(134.7)	0.51#
Bile leakage	34 (6.9%)	23 (4.6%)	0.10
Liver failure^21^	13 (2.6%)	7 (1.3%)	0.17
Postoperative ascites	31 (6.3%)	18 (3.6%)	0.03
Bleeding	14 (2.8%)	12 (2.4%)	0.56
Pulmonary complications*	53 (10.9%)	22 (4.4%)	< 0.001
Cardiovascular complications†	39 (8%)	23 (4.6%)	0.01
Cerebrovascular complications‡	3 (0.6%)	3 (0.6%)	>0.9
CCI score ≥ 26.2	230 (47.2%)	149 (29.7%)	< 0.001
90-day mortality	18 (3.6%)	6 (1.2%)	0.01
Length of ICU stay (days), median (i.q.r.)	1 (0–2)	1 (0–1)	0.01¶
Length of hospital stay (days), median (i.q.r.)	9 (6–15)	8 (5–12)	0.01¶
Readmission within 90 days	39 (8%)	20 (4%)	0.007

Values are *n* (%) unless stated otherwise. *Any complication affecting the respiratory system after anaesthesia and surgery, including atelectasis, pulmonary embolism, hypoxaemia, and respiratory failure. †Any complication affecting the cardiovascular system after anaesthesia and surgery, including arrythmias, myocardial infarction, pericarditis, and heart failure. ‡Any complication affecting the cerebrovascular system after anaesthesia and surgery, including stroke, transient ischaemic attack, cerebrovascular embolism, and intracranial haemorrhage. ASA, American Association of Anesthesiologists; i.q.r., interquartile range; BMI, body mass index; s.d., standard deviation; CCI, Comprehensive Complication Index; ICU, intensive care unit. §χ^2^ or Fisher’s exact test, except ¶Mann–Whitney *U* test and #*t* test.

### Analysis before PSM

There were more patients with an ASA grade of > II (*P* = 0.04) and BMI ≥ 30 kg/m^2^ (62 (12.7%) *versus* 46 (9.1%); *P* = 0.05) in the open group than the laparoscopic group (*Table 1*). The tumour location differed between the two groups (*P* < 0.01); lesions were more likely to be located in the posterosuperior segments I (11 (2.2%) *versus* 5 (0.9%)), VII and VIII (184 (38%) *versus* 148 (29.7%)) in the open group. More patients presented more than one tumour in the open group (median 1 (i.q.r. 1–2) *versus* 1 (1–1); *P* < 0.001). The type of primary tumour differed between groups (*P* < 0.001); a greater proportion of patients with hepatocellular carcinoma underwent laparoscopy (260 (51.8%) *versus* 190 (39.1%)). Partial and wedge resections were more common in the laparoscopy group (*P* < 0.001 and *P* = 0.01). The surgery was more complex in the open group (Iwate score ≥ 7: 245 (50%) *versus* 164 (32.7%); *P* < 0.001) (*Table 1*). The majority of patients with liver disease had Child–Pugh A disease (*Table S1*). During surgery, the total Pringle time was longer in the laparoscopic group (mean(s.d.) 24.4(39.2) *versus* 39.8(53.6) min). Intraoperative blood loss and transfusion amount were greater in the open group (*P* < 0.001).

### Postoperative morbidity before PSM

Overall the severe morbidity rate was 38.2%, and 230 patients (47.2%) had a CCI score of ≥ 26.2 in the open group, whereas 146 patients (29.3%) experienced severe morbidity in the laparoscopic group (*P* < 0.001). Pulmonary complications were more common in the open group (53 patients (10.9%) *versus* 22 (4.4%); *P* < 0.001). Ninety-day mortality rates were 3.6% in the open group (18 deaths) and 1.2% (6) in the laparoscopic group (*P* = 0.01) (*Table 2*). The main causes of death were liver failure, acute respiratory failure, systemic inflammatory response syndrome, and cardiovascular complications. Hospital stay and intensive care unit stay were longer (*P* = 0.01), and the readmission rate was higher, in the open group.

**Table 2:** Ninety-day mortality: causes of deaths before propensity score matching

	Open group (*n* = 487)	Laparoscopic group (*n* = 501)	*P*‡
90-day mortality	18 (3.6%)	6 (1.2%)	0.01
**Cause of death**			
Systemic inflammatory response syndrome	3 (16.6%)	0 (0%)	0.07
Acute respiratory failure	4 (22.2%)	1 (16.6%)	0.16
Liver failure^21^	7 (38.8%)	3 (50%)	0.18
Bleeding	2 (11.1%)	0 (0%)	0.15
Cardiovascular complications*	1 (5.5%)	2 (33.3%)	0.58
Cerebrovascular complications†	1 (5.5%)	0 (0%)	0.31

Values are *n* (%). *Any complication affecting the cardiovascular system after anaesthesia and surgery, including arrythmias, myocardial infarction, pericarditis, and heart failure. †Any complication affecting the cerebrovascular system after anaesthesia and surgery, including stroke, transient ischaemic attack, cerebrovascular embolism, and intracranial haemorrhage. ‡χ^2^ or Fisher’s exact test.

Independent risk factors associated with severe morbidity were an open approach (hazard ratio (HR) 1.59, 95% confidence interval (c.i.) 1.19 to 2.11; *P* < 0.001), Charlson Co-morbidity Index score > 7 (HR 1.69, 1.26 to 2.27; *P* < 0.001), more than one resected tumour (HR 1.55, 1.13 to 2.11; *P* = 0.006), major hepatectomy (HR 1.86, 1.22 to 2.83; *P* = 0.003), and Iwate score ≥ 7 (HR 1.43, 1.02 to 2.01; *P* = 0.03) (*Table 3*).

**Table 3:** Univariable and multivariable Cox regression analysis with robust estimator of severe morbidity after liver resection in the entire cohort (988 patients)

	No. with severe morbidity	Univariable analysis		Multivariable analysis	
		Hazard ratio*	*P*	Hazard ratio*	*P*
**Surgical technique**					
Open	230 (47.2%)	1.5 (1.34, 1.87)	< 0.001	1.59 (1.19, 2.11)	< 0.001
Laparoscopic	149 (29.7%)	1.00 (reference)		1.00 (reference)	
**Sex**			0.83		
Male	245 (64.6%)				
Female	134 (35.4%)				
**ASA fitness grade**		1.47 (1.13, 1.90)	0.003	1.19 (0.89, 1.58)	0.23
≤ II	161 (42.5%)				
> II	218 (57.5%)				
**BMI (kg/m^2^)**			0.26		
< 30	346 (91.3%)				
≥ 30	33 (8.7%)				
**Charlson Co-morbidity Index Score^17^**		1.91 (1.47, 2.48)	0.003	1.69 (1.26, 2.27)	< 0.001
≤ 7	156 (41.2%)				
> 7	223 (58.8%)				
**Performance status^18^**		1.72 (1.27, 2.33)	< 0.001	1.30 (0.92, 1.82)	0.12
≤ 1	273 (72%)				
> 1	106 (28%)				
**Liver disease**			0.50		
Yes	125 (33%)				
No	254 (67%)				
**Tumour in segment I**			0.79		
Yes	7 (1.8%)				
No	9 (98.2%)				
**Tumour in segment II–IV**			0.14		
Yes	139 (32.3%)				
No	230 (67.7%)				
**Tumour in segment V–VI**			0.14		
Yes	124 (42%)				
No	147 (58%)				
**Tumour in segment VII–VIII**			0.77		
Yes	119 (35.8%)				
No	213 (64.2%)				
**No. of tumours**		1.91 (1.47, 2.48)	< 0.001	1.55 (1.13, 2.11)	0.006
≤ 1	254 (67%)				
> 1	125 (33%)				
**Hepatocellular carcinoma**			0.32		
Yes	165 (36.7%)				
No	285 (63.3%)				
**Cholangiocarcinoma**			0.06		
Yes	51 (46.8%)				
No	56 (53.2%)				
**Colorectal metastases**			0.78		
Yes	131 (37.8%)				
No	215 (62.2%)				
**Other tumours**			> 0.9		
Yes	28 (7.4%)				
No	57 (92.6%)				
**Partial resection**		0.44 (0.44, 0.58)	< 0.001	1.10 (0.72, 1.68)	0.64
Yes	109 (28.8%)				
No	270 (71.2%)				
**Left lateral sectionectomy**			0.14		
Yes	19 (5%)				
No	360 (95%)				
**Segmentectomy**			0.4		
Yes	97 (25.6%)				
No	282 (74.4%)				
**Sectionectomy and more**		2.41 (1.82, 3.20)	< 0.001	1.15 (0.76, 1.74)	0.49
Yes	151 (39.8%)				
No	228 (60.2%)				
**Wedge resection**		0.51 (0.39, 0.66)	< 0.001	0.69 (0.48, 1.01)	0.05
Yes	126 (33.3%)				
No	253 (66.7%)				
**Anatomical resection**		2.15 (1.64, 2.82)	< 0.001	0.96 (0.63, 1.46)	0.87
Yes	258 (68.4%)				
No	121 (31.6%)				
**Major hepatectomy**		2.78 (2.08, 3.71)	< 0.001	1.86 (1.22, 2.83)	0.003
Yes	149 (39.3%)				
No	230 (60.7%)				
**Iwate score**		2.43 (1.84, 3.20)	< 0.001	1.43 (1.02, 2.01)	0.03
< 7	217 (57.3%)				
≥ 7	162 (42.7%)				

Values are *n* (%) unless otherwise stated; *values in parentheses are 95% confidence intervals. ASA, American Society of Anesthesiologists.

### Analysis after PSM

After PSM, 294 of 487 patients in the open group could be matched with 294 of 501 patients in the laparoscopic group. All clinically relevant factors were balanced between the two groups. There was no difference in demographic or operative characteristics between the groups after PSM (*Table 4*).

**Table 4:** Patient characteristics in open and laparoscopic groups after propensity score matching

	Open group (*n* = 294)	Laparoscopic group (*n* = 294)	*P*§
**Patient characteristics**			
Age (years), median (i.q.r.)	82 (81–83)	82 (81–84)	0.77¶
Sex			0.08
Male	201 (68.3%)	181 (61.5%)	
Female	93 (31.4%)	113 (38.5%)	
ASA fitness grade, median (i.q.r.)	II (I–II)	II (I–II)	0.45¶
BMI ≥ 30 kg/m^2^	33 (11.2%)	27 (9.1%)	0.27
Charlson Co-morbidity Index score, median (i.q.r.)^17^	7 (5–10)	7 (6–10)	0.68¶
Performance status > 1^18^	63 (21.4%)	52 (17.7%)	0.29
**Tumour characteristics**			
Tumour location (liver segment)			0.76
I	8 (2.7%)	5 (1.7%)	
II–IV	107 (36.4%)	109 (37.1%)	
V–VI	79 (26.8%)	82 (27.9%)	
VII–VIII	100 (34.1%)	98 (33.3%)	
No. of tumours, median (i.q.r.)	1 (1–1)	1 (1–1)	0.73¶
Type of primary tumour			0.38
Hepatocellular carcinoma	123 (41.8%)	140 (47.6%)	
Colorectal liver metastases	117 (39.8%)	112 (38.1%)	
Cholangiocarcinoma	29 (9.9%)	20 (6.8%)	
Others	25 (8.5%)	22 (7.5%)	
**Surgical characteristics**			
Type of resection			0.44
Partial	126 (42.9%)	133 (45.4%)	0.56
Left lateral sectionectomy	14 (4.8%)	16 (5.5%)	0.70
Segmentectomy	70 (23.9%)	78 (26.6%)	0.44
Sectionectomy and more	84 (28.6%)	67 (22.8%)	0.10
Wedge resection	135 (45.9%)	135 (45.9%)	> 0.99
Anatomical resection	166 (56.5%)	151 (51.4%)	0.28
Major hepatectomy	63 (21.4%)	63 (21.4%)	> 0.99
Iwate score ≥ 7	125 (42.5%)	119 (40.5%)	0.17
**Outcomes**			
Total Pringle time (min), mean(s.d.)	23.2(39.1)	37.1(52.5)	0.001#
Intraoperative blood loss (ml), mean(s.d.)	476.1(492.4)	275.3(454.1)	< 0.001#
Intraoperative blood transfusion	45 (15.5%)	32 (11%)	0.13
Conversion to open surgery	–	25 (8.5%)	
Duration of operation (min), mean(s.d.)	255.4(122.1)	279.9(143.9)	0.08#
Bile leakage	12 (4.2%)	14 (4.9%)	0.84
Liver failure^21^	7 (2.5%)	6 (2.1%)	> 0.99
Postoperative ascites	21 (7.4%)	15 (5.3%)	0.38
Bleeding	8 (2.8%)	8 (2.8%)	> 0.99
Pulmonary complications*	36 (12.2%)	15 (5.1%)	0.003
Cardiovascular complications†	23 (8.1%)	13 (4.6%)	0.12
Cerebrovascular complications‡	2 (0.7%)	1 (0.4%)	> 0.99
CCI score ≥ 26.2	138 (46.9%)	104 (35.3%)	0.006
90-day mortality	12 (4%)	4 (1%)	0.04
Length of ICU stay (days), median (i.q.r.)	1 (0–2)	1 (0–1)	0.04¶
Length of hospital stay (days), median (i.q.r.)	9 (6–14)	8 (5–13)	0.02¶
Readmission within 90 days	26 (9%)	10 (3.4%)	0.008

Values are *n* (%) unless stated otherwise. *Any complication affecting the respiratory system after anaesthesia and surgery, including atelectasis, pulmonary embolism, hypoxaemia, and respiratory failure. †Any complication affecting the cardiovascular system after anaesthesia and surgery, including arrythmias, myocardial infarction, pericarditis, and heart failure. ‡Any complication affecting the cerebrovascular system after anaesthesia and surgery, including stroke, transient ischaemic attack, cerebrovascular embolism, and intracranial haemorrhage. ASA, American Association of Anesthesiologists; i.q.r., interquartile range; BMI, body mass index; s.d., standard deviation; CCI, Comprehensive Complication Index; ICU, intensive care unit. §χ^2^ or Fisher’s exact test, except ¶Mann–Whitney *U* test and #*t* test.

### Postoperative morbidity after PSM

The total Pringle time was longer in the laparoscopic group (mean(s.d.) 23.2(39.1) *versus* 37.1(52.5) min; *P* = 0.001). Intraoperative blood loss was greater in the open group (*P* = 0.001). The pulmonary complication rate was higher in the open group (36 (12.2%) *versus* 15 (5.1%); *P* = 0.003). The CCI score was ≥ 26.2 in 138 patients (46.9%) in the open group *versus* 104 (35.3%) in the laparoscopic group (*P* = 0.006). Ninety-day mortality rates were higher and intensive care unit stays longer in the open group (*P* = 0.04). Length of hospital stay and readmission rate were also higher in the open group (*Table 4*).

## Discussion

This analysis of a large multicentre series comparing open and laparoscopic hepatectomies in patients aged > 80 years found that those in the laparoscopic group had fewer complications. After multivariable and PSM analysis, open surgery was associated with an increased risk of pulmonary complications, CCI score ≥ 26.2, 90-day mortality, longer intensive care unit and hospital stay, and readmission.

Laparoscopic liver resection has been associated with less morbidity than open operation^2,27^. The literature lacks robust evidence regarding similar outcomes in patients aged > 80 years^28–38^. Elderly patients are generally defined as those aged > 65 years^39–41^, but many researchers preferred to use 70 years as the cut-off^42,43^. Very few studies have analysed patients aged > 80 years as a separate subset, and the reported studies were small^6,7,30–38,42–44^.

Although 324 patients (32.7%) recruited in this study presented with liver disease, this was not an independent prognostic factor for morbidity. Clearly, any octogenarian or nonagenarian considered for surgery has good performance status. In the present analysis, some 80% of patients had a performance status of 1.

In the field of laparoscopic abdominal surgery compared with open operation, the first three meta-analyses^6–8^ concerning octogenarian patients favoured laparoscopy more. The literature is confusing regarding the benefits of liver resection for patients > 80 years old, who were included in few studies^8–15^, and in most cases only in the open group. Martínez-Cecilia *et al.*^44^ observed that the benefits of the laparoscopic approach appeared to wane with increasing age, with no benefits in octogenarians except for a shorter intensive care unit stay. Later, Nomi *et al.*^45^ compared laparoscopic *versus* open surgery for hepatocellular carcinoma in elderly patients, observing better short-term outcomes in those aged ≥ 75 years in the laparoscopic group. However, operating time, which is key in the elderly population, was not reported. Sijberden *et al.*^30^ compared minimally invasive *versus* open liver resection for hepatocellular carcinoma in the elderly. No differences were observed between groups regarding morbidity rates, probably owing to the small sample size (59 patients).

In the present study, laparoscopy was associated with fewer pulmonary complications before and after PSM (from 12.2% in the open group to 5.1% in the laparoscopic group after PSM). Pulmonary complications account for the majority of morbidity following abdominal surgery, likely due to the altered respiratory physiology associated with the disruption of the abdominal musculature^46–48^. Lastly, 90-day mortality is key for elderly patients, as there is less long-term survival than for younger patients. In that respect, Mueller *et al.*^48^ analysed 2397 elderly patients who underwent open hepatic resection. In unadjusted analysis, patients ≥ 80 years of age had higher rates of 90-day mortality (13.3 *versus* 3.6%; *P* < 0.001). In the present study, the 90-day postoperative mortality rate was below 4% with the open approach and below 2% in the laparoscopic group, probably because all the participating centres were high-volume units, reporting low mortality rates, and the study period was after 2014, so the laparoscopic liver resection learning curve had been completed.

Some limitations of the present study warrant discussion. The study was retrospective, so prone to information bias, and the patients were not assigned randomly, so some selection bias could be present despite the adjusted and matched methods of analysis. A randomized clinical trial evaluating the impact of this approach on outcomes would be difficult to design on ethical grounds. PSM, the most robust statistical approach in a retrospective study, was adopted. Although this report provides complete data on a larger cohort than would have been possible at a single centre, the follow-up protocols and management approach used may have differed between hospitals. Even given the weaknesses of the study, this is the largest study comparing the laparoscopic approach for elderly patients *versus* open hepatectomy, and has shown that laparotomy could be associated with an increased risk of morbidity and 90-day mortality in patients aged > 80 years.

In conclusion, the laparoscopic approach for elderly patients offers better morbidity and mortality outcomes than the open approach, and should be considered as a default option, given the superior results observed in this population.

## Supplementary Material

zraf102_Supplementary_Data

## Data Availability

The data that support the findings of this study are available on request from the corresponding author (C.G.G.).
