# Supplementary material for: Laparoscopic versus open liver resection in patients aged at least 80 years: retrospective propensity score-matched cohort study
Source: BJS Open. 2025 Nov 28;9(6):zraf102. doi: 10.1093/bjsopen/zraf102 (PMC12662231; doi:10.1093/bjsopen/zraf102)
Supplement: zraf102_Supplementary_Data [file zraf102_supplementary_data.zip › Supplementary_Material.docx]

#### **Laparoscopic versus Open Liver Resection in patients over 80 years old: retrospective propensity score-matched cohort study**

**Concepción Gómez-Gavara^1*^, MD, PhD, Zeniche Morise^2*^, MD, PhD, Victor López-López^3^, MD, Christoph Kuemmerli^4^, MD, Daniel Esono^5^, MD, Kazuharu Igarashi^6^, MD, Kohei Mishima^6^, MD, Akishige Kanazawa^7^, MD, Shogo Tanaka^8^, MD, PhD, Shoji Kubo^8^, MD, PhD, Satoshi Nemoto^9^, MD, Goro Honda^9^, MD, PhD, Kazuteru Monden^10^, MD, FACS, Masaki Ueno^11^, MD, PhD, Yasuhito Iwao^12^, MD, Naoto Gotohda^13^, MD, PhD, Masashi Kudo^13^, MD, Hiroyuki Nitta^14^, MD, PhD, Satoshi Amano^14^, MD, PhD, Rafael Díaz-Nieto^15^, MD, PhD, Alex Gordon-Weeks^15^, MBChB, BSc, MRCS,** **Serena Langella^16^, MD, Alessandro Ferrero^16^, MD, PhD, Yuichiro Otsuka^17^, MD, PhD, Hironori Kaneko^17^, MD, PhD, Riccardo Boetto^18^, MD, Umberto Cillo^18^, MD, Daniel D´Souza^19^, MD, Pablo E. Serrano^19^, MD, PhD, Giammauro Berardi^20^, MD, PhD, Marco Angrisani^20^, MD, Giuseppe Maria Ettorre^20^, MD, Parissa Tabrizian^21^, MD, Allen Yu^21^, MD, PhD, Brian K. P. Goh^22^, MBBS, MMed, MSc, FRCS, Takuya Minagawa^23^, MD, Osamu Itano^23^,** **MD, PhD, Daisuke Asano^24^, MD, PhD, Minoru Tanabe^24^, MD, PhD, Marcello Di Martino^25^, MD, PhD, Elena Martín-Pérez^25^, MD, PhD, Simone Famularo^26^, MD, Elisa Paoluzzi Tomada^26^, MD, Guido Torzilli^26^, MD, PhD, Jaime Arthur Pirola Krüger^27^, MD, Paulo Herman^27^, MD, Mario Giuffrida^28^, MD, PhD, Ramon Charco^1^, MD, PhD, Mikel Gastaca^29^, MD, PhD, Waclaw Holowko^30^, MD, PhD, Stephanie Truant^31^, MD, PhD, Kit-Man Ho^32^, MD, Kai-Chi Cheng^32^, MD, Rafael José Maurette^33^, MD, Laura-Ann Blatt^34^, MD, Tatiana Belda^35^, MD, PhD, Yuta Abe^36^ MD, PhD,**  [**Shuichir**](https://pubmed.ncbi.nlm.nih.gov/?term=Uemura+S&cauthor_id=31271511)**o Uemura^36^ MD, PhD,** **and Go Wakabayashi^5^ MD, PhD.**

Affiliations:

1. Autonomous University of Barcelona, Spain. HBP and Transplantation Surgery Service. Vall d´Hebron University Hospital. Vall d´Hebron Institute of research.
2. Department of Surgery, Fujita Health University School of Medicine Okazaki Medical Center, Okazaki 444-0827, Aichi, Japan.
3. Department of General, Visceral and Transplantation Surgery, Clinic and University Hospital Virgen de La Arrixaca, IMIB-ARRIXACA, El Palmar, Murcia, Spain.
4. Department of Surgery, Clarunis University Center for Gastrointestinal and Liver Disease, St. Clara Hospital and University Hospital, Kleinriehenstrasse 30, 4058, Basel, Switzerland.
5. Department of Information and Communications Technologies, Universitat Pompeu Fabra, Barcelona, Spain.
6. Department of Surgery, Ageo Central General Hospital, Ageo, Japan.
7. Department of Hepato-Biliary-Pancreatic Surgery, Osaka City General Hospital, Miyakojima-ku, Osaka, Japan.
8. Department of Hepato-Biliary-Pancreatic Surgery, Osaka City University
   Graduate School of Medicine, Japan.
9. Department of Surgery, Institute of Gastroenterology, Tokyo Women’s Medical University, 8-1 Kawada Cho, Shinjyuku-ku, Tokyo, Japan.
10. Department of Surgery, Fukuyama city hospital, Japan.
11. Department of Surgery, Wakayama Medical University, Japan.
12. Department of Surgery, Ohta-Nishinouchi Hospital, Japan.
13. Department of Surgery, National Cancer Center Hospital East, Japan.
14. Department of Surgery, Iwate Medical University, Japan.
15. Department of Surgery, Liverpool University Hospital, United Kingdom.
16. S. Chirurgia Generale e Oncologica, Ospedale Mauriziano, Italy.
17. Department of Surgery, Toho University Faculty of Medicine, Tokyo, Japan.
18. Chirurgia Epatobiliare e dei Trapianti Epatici, Padova, Italy.
19. McMaster University, Hamilton, ON, Canada.
20. POIT- SCF General Surgery and Transplant Unit, Rome, Italy.
21. Mount Sinai Liver Cancer Program, Tisch Cancer Institute, Icahn School of Medicine at Mount Sinai, New York, New York, USA.
22. Department of Hepatopancreatobiliary and Transplant Surgery, Singapore General Hospital and National Cancer Centre Singapore, Singapore. Surgery Academic Clinical Program, Duke-National University of Singapore Medical School, Singapore.
23. Department of Hepato-Biliary-Pancreatic and Gastrointestinal Surgery, School of Medicine, International University of Health and Welfare, Chiba, Japan.
24. Department of Hepatobiliary and Pancreatic Surgery, Graduate School of Medicine, Tokyo Medical and Dental University, Tokyo, Japan.
25. HepatoPancreatoBiliary Unit, Department of General and Digestive Surgery, Hospital Universitario La Princesa, Instituto deInvestigación Sanitaria Princesa (IIS-IP), Universidad Autónoma de Madrid (UAM).
26. Department of Biomedical Sciences, Humanitas University, Pieve Emanuele, Milan, Italy.
27. Department of Gastroenterology, Hospital das Clinicas, University of São Paulo Medical School, Brazil.
28. General Surgery Unit, Parma University Hospital, Italy.
29. Hepatobiliary Surgery and Liver Transplant Unit, Cruces University Hospital BioCruces Health Research Institute, Vizcaya, Spain.
30. Department of General, Transplant and Liver Surgery, Medical University of Warsaw, Poland.
31. Department of Digestive Surgery and Liver Transplantation, University Hospital C. Huriez, Lille, France.
32. Department of Surgery, Kwong Wah Hospital, Hong Kong.
33. General Surgery Department, British Hospital of Buenos Aires, Argentina.
34. Klinikum Saarbrücken gGmbH, Winterberg 1, 66119 Saarbrücken, Germany.
35. Department of Surgery, Verge dels Lliris Hospital, Alcoy, Spain.
36. Department of Surgery, Keio University School of Medicine, Keio University Hospital,

35 Shinano-machi Shinjuku-ku Tokyo, Japan.

***Corresponding author/reprint requests:**

Zenichi Morise M.D., Ph.D., FACS

Professor and Chairman, Department of Surgery Fujita Health University School of Medicine

Founding Past Director, Fujita Health University Okazaki Medical Center

Program Officer, Japan Agency for Medical Research and Development

Deputy Chief Editor, Fujita Medical Journal

1 Gotanda Harisakicho,

Okazaki, AICHI 444-0827 JAPAN

Email: zmorise@gmail.com

Phone:+81-564-64-8800 FAX:+81-564-64-8135

Co-corresponding: Concepción Gómez-Gavara, PhD

Consultant surgeon in HBP and Liver Transplantation PhD Vall D´Hebron University Hospital, Barcelona, SPAIN

Passeig de la Vall d´Hebron, 119-129, 08035, Barcelona.

Email: concepcion.gomez@vallhebron.cat

Phone: Office: +34932746000. Secretary: +34932746113. Fax: +34932746112.

Twitter: @GavaraGomez

**Supplementary Materials - Index**

| **Supplementary Methods** |  |
| --- | --- |
| Reporting data | *Page 5* |
|  |  |
| **Supplementary Figures and Tables** |  |
| Supplementary figure 1. Histogram of the patient´s distribution per centre. | *page 5* |
| Supplementary table 1. Tumour characteristics in liver disease cases. | *page 5* |
| **References** | *page 6-11* |
|  |  |

**Supplementary Methods**

Confidentiality and anonymity were always maintained. In the event of transfer of encrypted data outside the EU to entities in the present group, service providers or scientific researchers, the data was protected with safeguards such as contracts or other mechanisms established by the data protection authorities. The respective agreements/contracts for the transfer of data to countries outside the EU were made.

The local investigator was responsible for recording and reporting all complications, regardless of causality, observed in all patients included.

Access to the anonymous data was restricted to the local investigator or the local data manager.

**Supplementary Figures and Tables**

Supplementary figure 1. Histogram of the patient´s distribution per centre.


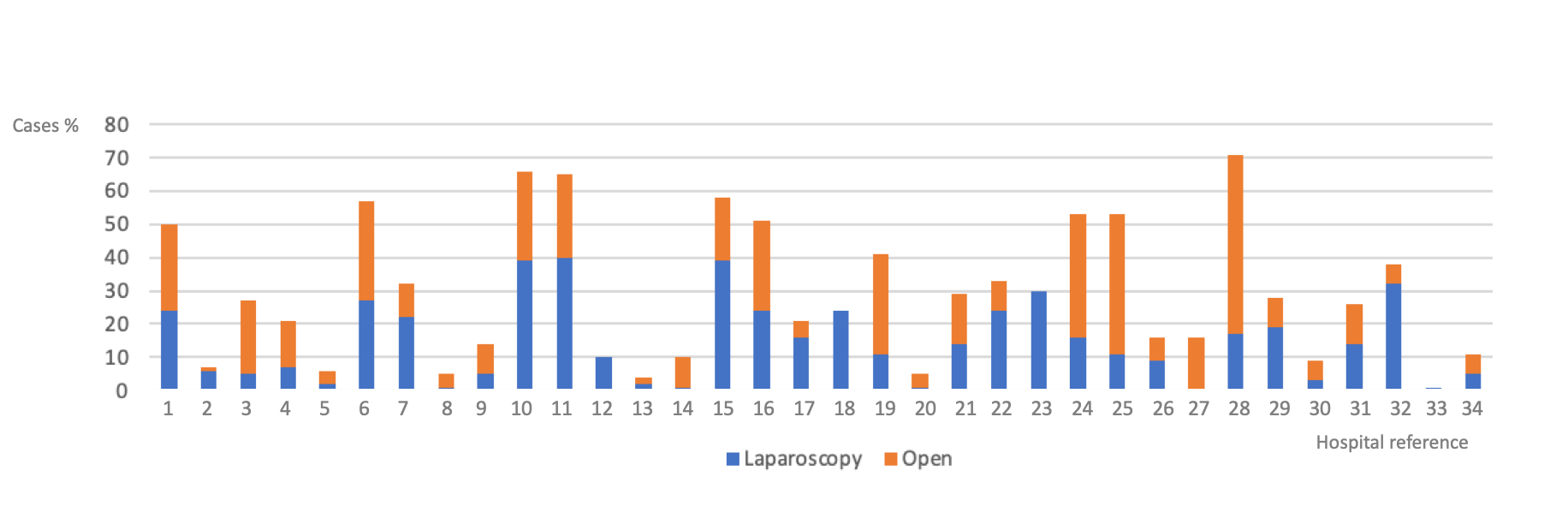


Supplementary table 1. Tumour characteristics in liver disease cases.

|  | **Open group patients** | **Laparoscopic group patients** | ***p-value*** |
| --- | --- | --- | --- |
| Variable | **n = 487** | **n = 501** |  |
| ***Tumor Characteristics*** |  |  |  |
| Hepatocellular carcinoma | 190 (39.1%) | 260 (51.8%) | p=0.01 |
| Child Pugh A | 174 (91.5%) | 219 (84.2%) |  |
| Child Pugh B | 16 (8.5%) | 41 (15.8%) |  |
| Cholangiocarcinoma | 76 (15.6%) | 31 (6.2%) | p=0.29 |
| Child Pugh A | 57 (75%) | 20 (64.5%) |  |
| Child Pugh B | 19 (25%) | 11 (35.5%) |  |
|  |  |  |  |
|  |  |  |  |
|  |  |  |  |
|  |  |  |  |
|  |  |  |  |

**References**

1. Dindo D, Demartines N, Clavien P-A. Classification of surgical complications: a new proposal with evaluation in a cohort of 6336 patients and results of a survey. Ann Surg. 2004;240(2):205–13.
2. Charlson ME, Pompei P, Ales K L. A new method of classifying prognostic comorbidity in longitudinal studies: development and validation. J Chronic Dis. 1987;40(5):373-83.
3. Wakabayashi G, Cherqui D, Geller DA, Buell JF, Kaneko H, Han HS, et al. Recommendations for laparoscopic liver resection: a report from the second international consensus conference held in Morioka. Ann Surg. 2015 Apr;261(4):619–29.
4. Fretland ÅA, Dagenborg VJ, Bjørnelv GMW, Kazaryan AM, Kristiansen R, Fagerland MW, et al. Laparoscopic Versus Open Resection for Colorectal Liver Metastases: The OSLO COMET Randomized Controlled Trial. Ann Surg. 2018;267(2):199–207.
5. Bates AT, Divino C. Laparoscopic surgery in the elderly: a review of the literature. Aging Dis. 2015 Mar;6(2):149–55.
6. Wirth S, Biesemann A, Spaeth J, Schumann S. Pneumoperitoneum deteriorates intratidal respiratory system mechanics: an observational study in lung-healthy patients. Surg Endosc. 2017;31(2):753–60.
7. Chesney T, Acuna SA. Do elderly patients have the most to gain from laparoscopic surgery? Ann Med Surg. 2015 Sep;4(3):321–3.
8. Li Y, Wang S, Gao S, Yang C, Yang W, Guo S. Laparoscopic colorectal resection versus open colorectal resection in octogenarians: a systematic review and meta-analysis of safety and efficacy. Tech Coloproctol. 2016 Mar;20(3):153–62.
9. Xie M, Qin H, Luo Q, He X, Lan P, Lian L. Laparoscopic Colorectal Resection in Octogenarian Patients: Is it Safe? A Systematic Review and Meta-Analysis. Medicine (Baltimore). 2015 Oct;94(42):e1765.
10. Xie S-M, Xiong J-J, Liu X-T, Chen H-Y, Iglesia-García D, Altaf K, et al. Laparoscopic Versus Open Liver Resection for Colorectal Liver Metastases: A Comprehensive Systematic Review and Meta-analysis. Sci Rep. 2017;7(1):1012.
11. Cheung TT, Poon RTP, Yuen WK, Chok KSH, Tsang SHY, Yau T, et al. Outcome of laparoscopic versus open hepatectomy for colorectal liver metastases. ANZ J Surg. 2013 Nov;83(11):847–52.
12. Jung KU, Kim HC, Cho YB, Kwon CHD, Yun SH, Heo JS, et al. Outcomes of simultaneous laparoscopic colorectal and hepatic resection for patients with colorectal cancers: a comparative study. J Laparoendosc Adv Surg Tech A. 2014 Apr;24(4):229–35.
13. Takasu C, Shimada M, Sato H, Miyatani T, Imura S, Morine Y, et al. Benefits of simultaneous laparoscopic resection of primary colorectal cancer and liver metastases. Asian J Endosc Surg. 2014 Jan;7(1):31–7.
14. de’Angelis N, Eshkenazy R, Brunetti F, Valente R, Costa M, Disabato M, et al. Laparoscopic versus open resection for colorectal liver metastases: a single-center study with propensity score analysis. J Laparoendosc Adv Surg Tech A. 2015 Jan;25(1):12–20.
15. Hasegawa Y, Nitta H, Sasaki A, Takahara T, Itabashi H, Katagiri H, et al. Long-term outcomes of laparoscopic versus open liver resection for liver metastases from colorectal cancer: A comparative analysis of 168 consecutive cases at a single center. Surgery. 2015 Jun;157(6):1065–72.
16. Langella S, Russolillo N, D’Eletto M, Forchino F, Lo Tesoriere R, Ferrero A. Oncological safety of ultrasound-guided laparoscopic liver resection for colorectal metastases: a case-control study. Updates Surg. 2015 Jun;67(2):147–55.
17. Ratti F, Catena M, Di Palo S, Staudacher C, Aldrighetti L. Impact of totally laparoscopic combined management of colorectal cancer with synchronous hepatic metastases on severity of complications: a propensity-score-based analysis. Surg Endosc. 2016;30(11):4934–45.
18. De Cassai A, Boscolo A, Tonetti T, Ban I, Ori C. Assignment of ASA-physical status relates to anesthesiologists' experience: a survey-based national-study. Korean J Anesthesiol. 2019 Feb;72(1):53-59.
19. Oken MM, Creech RH, Tormey DC, Horton J, Davis TE, et al. Toxicity and response

criteria of the Eastern Cooperative Oncology Group. American Journal of Clinical Oncology. 1982; 5(6), 649-655.

1. European Association For The Study Of The Liver, European Organisation For Research and Treatment of Cancer. EASLEORTC clinical practice guidelines: management of hepatocellular carcinoma. J Hepatol 2012; 56: 908-943.
2. Couinaud C.
3. [Balzan](https://pubmed.ncbi.nlm.nih.gov/?term=Balzan+S&cauthor_id=16327492) S, [Belghiti](https://pubmed.ncbi.nlm.nih.gov/?term=Belghiti+J&cauthor_id=16327492) J, [Farges](https://pubmed.ncbi.nlm.nih.gov/?term=Farges+O&cauthor_id=16327492) O, [Ogata](https://pubmed.ncbi.nlm.nih.gov/?term=Ogata+S&cauthor_id=16327492) S, [Sauvanet](https://pubmed.ncbi.nlm.nih.gov/?term=Sauvanet+A&cauthor_id=16327492) A, et al. The "50-50 criteria" on postoperative day 5: an accurate predictor of liver failure and death after hepatectomy. Ann Surg . 2005 Dec;242(6):824-8, discussion 828-9.
4. Dindo D, Demartines N, Clavien P-A. Classification of surgical complications: a new proposal with evaluation in a cohort of 6336 patients and results of a survey. Ann Surg. 2004;240(2):205–13.
5. Yamashita S, Sheth RA, Niekamp AS, Aloia TA, Chun YS, Lee JE, et al. Comprehensive Complication Index Predicts Cancer-specific Survival After Resection of Colorectal Metastases Independent of RAS Mutational Status. Ann Surg. 2017 Dec;266(6):1045–54.
6. World Medical Association Declaration of Helsinki: Ethical Principles for Medical Research Involving Human Subjects, 2008.
7. Rubin DB, Thomas N. Matching using estimated propensity scores: relating theory to practice. Biometrics. 1996 Mar;52(1):249–64.
8. Rosenbaum PR, Rubin DB. Constructing a Control Group Using Multivariate Matched Sampling Methods That Incorporate the Propensity Score. Am Stat. 1985 Feb;39(1):33.
9. Pilgrim CHC, To H, Usatoff V, Evans PM. Laparoscopic hepatectomy is a safe

procedure for cancer patients. HPB (Oxford). 2009 May;11(3):247–51.

1. Chan ACY, Poon RTP, Cheung TT, Chok KSH, Dai WC, Chan SC, et al. Laparoscopic versus open liver resection for elderly patients with malignant liver tumors: A single-center experience. J Gastroenterol Hepatol. 2014 Jun;29(6):1279–83.
2. Wang X-T, Wang H-G, Duan W-D, Wu C-Y, Chen M-Y, Li H, et al. Pure Laparoscopic Versus Open Liver Resection for Primary Liver Carcinoma in Elderly Patients. Medicine (Baltimore). 2015 Oct;94(43):e1854.
3. Sijberden JP, Cipriani F, Lanari J, Russolillo N, Benedetti Cacciaguerra A, Osei Bordom D, et al. Minimally invasive ver-sus open liver resection for hepatocellular carcinoma in the elderly: international multicentre propensity score-matched study. Br J Surg. 2023 Jul 17;110(8):927–30.
4. Amato B, Aprea G, De Rosa D, Milone M, di Domenico L, Amato M, et al. Laparoscopic hepatectomy for HCC in elderly patients: risks and feasibility. Aging Clin Exp Res. 2017 Feb;29(Suppl 1):179–83.
5. Zeng Y, Tian M. Laparoscopic versus open hepatectomy for elderly patients with liver metastases from colorectal cancer. J BUON. 2016;21(5):1146–52.
6. Badawy A, Seo S, Toda R, Fuji H, Fukumitsu K, Ishii T, et al. A Propensity Score-Based Analysis of Laparoscopic Liver Resection for Liver Malignancies in Elderly Patients. J Invest Surg. 2019 Jan;32(1):75–82.
7. Goh BKP, Chua D, Syn N, Teo J-Y, Chan C-Y, Lee S-Y, et al. Perioperative Outcomes of Laparoscopic Minor Hepatectomy for Hepatocellular Carcinoma in the Elderly. World J Surg. 2018 Dec;42(12):4063–9.
8. Delvecchio A, Conticchio M, Ratti F, Gelli M, Anelli FM, Laurent A, et al. Laparoscopic major hepatectomy for hepatocellular carcinoma in elderly patients: a multicentric propensity score‑based analysis. Surg Endosc. 2021 Jul;35(7):3642–52.
9. Dumronggittigule W, Han H-S, Ahn S, Yoon Y-S, Cho JY, Choi Y. Laparoscopic versus Open Hepatectomy for Hepatocellular Carcinoma in Elderly Patients: A Single-Institutional Propensity Score Matching Comparison. Dig Surg. 2020;37(6):495–504.
10. Kim JM, Kim S, Rhu J, Choi G-S, Kwon CHD, Joh J-W. Elderly Hepatocellular Carcinoma Patients: Open or Laparoscopic Approach? Cancers (Basel). 2020 Aug 14;12(8).
11. Monden K, Sadamori H, Hioki M, Ohno S, Takakura N. Short-term outcomes of laparoscopic versus open liver resection for hepatocellular carcinoma in older patients: a propensity score matching analysis. BMC Surg. 2022 Feb 23;22(1):63.
12. Gómez Gavara C, Esposito F, Gurusamy K, Salloum C, Lahat E, Feray C, et al. Liver transplantation in elderly patients: a systematic review and first meta-analysis. HPB (Oxford). 2018 Aug 23;
13. Gómez-Gavara C, Lim C, Adam R, Zieniewicz K, Karam V, Mirza D, et al. The impact of advanced patient age in liver transplantation: a European Liver Transplant Registry propensity-score matching study. HPB (Oxford). 2022 Jun;24(6):974–85.
14. Gómez-Gavara C, Charco R, Tapiolas I, Ridaura N, Campos-Varela I, Dopazo C, et al. Recipient Age for Liver Transplantation: Should It Be Limited? A Propensity Score Matching Analysis of a Large European Series. Transplant Proc. 2020 Jun;52(5):1442–9.
15. Martínez-Cecilia D, Cipriani F, Vishal S, Ratti F, Tranchart H, Barkhatov L, et al. Laparoscopic Versus Open Liver Resection for Colorectal Metastases in Elderly and Octogenarian Patients: A Multicenter Propensity Score Based Analysis of Short- and Long term Outcomes. Ann Surg. 2017;265(6):1192–200.
16. Di Martino M, Dorcaratto D, Primavesi F, Syn N, Blanco-Terés L, Dupré A, et al. Liver resection in elderly patients with extensive CRLM: Are we offering an adequate treatment? A propensity score matched analysis. Eur J Surg Oncol. 2022 Jun;48(6):1331–8.
17. Iswanto S, Cheek S, Tsung A, Marsh JW, Geller D. Minimally invasive liver resection for primary and metastatic liver tumors: influence of age on perioperative complications and mortality. Surg Endosc (2018) 32:1885–1891.
18. Nomi T, Hirokawa F, Kaibori M, Ueno M, Tanaka S, Hokuto D, et al. Laparoscopic versus open liver resection for hepatocellular carcinoma in elderly patients: a multi-centre propensity score-based analysis. Surg Endosc. 2019 May 15.
19. Nobili C, Marzano E, Oussoultzoglou E, Rosso E, Addeo P, Bachellier P, et al. Multivariate analysis of risk factors for pulmonary complications after hepatic resection. Ann Surg. 2012 Mar;255(3):540–50.
20. Fuks D, Cauchy F, Ftériche S, Nomi T, Schwarz L, Dokmak S, et al. Laparoscopy Decreases Pulmonary Complications in Patients Undergoing Major Liver Resection. Ann Surg. 2016 Feb;263(2):353–61.
21. Mueller JL, Molina G, Ferrone CR, Chang DC, Vagefi P, Tanabe KK, et al. Open hepatic resection in the elderly at two tertiary referral centers. Am J Surg. 2021 Sep;222(3):594–8.
