# Supplementary figures and images for: Laparoscopic versus open liver resection in patients aged at least 80 years: retrospective propensity score-matched cohort study
Source: BJS Open. 2025 Nov 28;9(6):zraf102. doi: 10.1093/bjsopen/zraf102 (PMC12662231; doi:10.1093/bjsopen/zraf102)

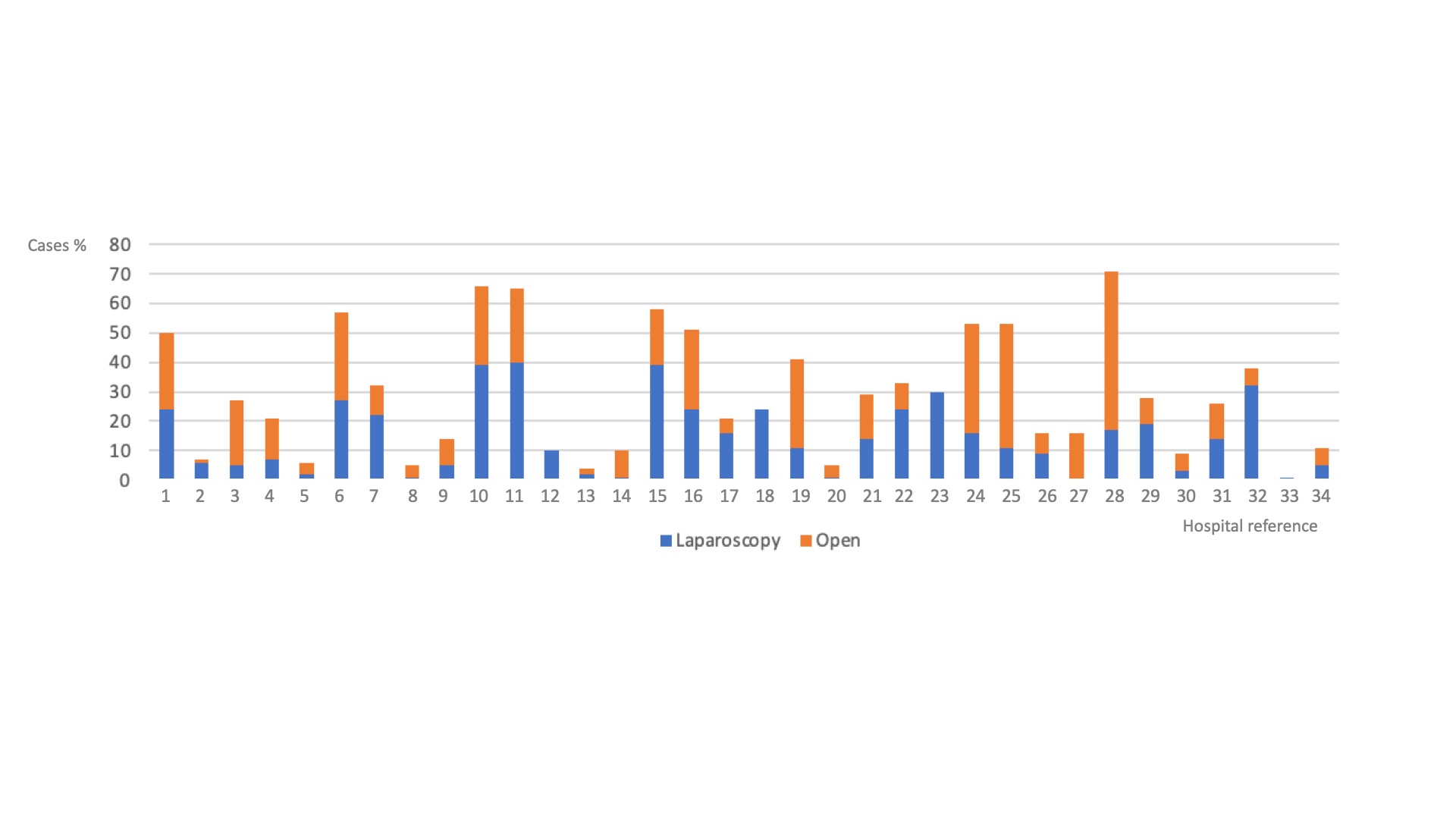

Supplement: zraf102_Supplementary_Data [file zraf102_supplementary_data.zip › FigureS1_BJS.jpeg]
